# High minimum inhibitory concentration of imipenem as a predictor of fatal outcome in patients with carbapenem non-susceptible *Klebsiella pneumoniae*

**DOI:** 10.1038/srep32665

**Published:** 2016-09-02

**Authors:** Ping-Feng Wu, Chien Chuang, Chin-Fang Su, Yi-Tsung Lin, Yu-Jiun Chan, Fu-Der Wang, Yin-Ching Chuang, L. Kristopher Siu, Chang-Phone Fung

**Affiliations:** 1 Division of Infectious Diseases, Department of Medicine, Taipei Veterans General Hospital, Taipei, Taiwan; 2 School of Medicine, National Yang-Ming University, Taipei, Taiwan; 3 Department of Medicine, Taipei Veterans General Hospital, Taipei, Taiwan; 4 Institute of Emergency and Critical Care Medicine, National Yang-Ming University, Taipei, Taiwan; 5 Division of Microbiology, Department of Pathology and Laboratory Medicine, Taipei Veterans General Hospital, Tainan, Taiwan; 6 Department of Internal Medicine and Medical Research, Chi Mei Medical Center, Tainan, Taiwan; 7 Institute of Infectious Diseases and Vaccinology, National Health Research Institutes, Miaoli, Taiwan; 8 Division of Infectious Diseases, Sijhih Cathy General Hospital, New Taipei City, Taiwan.

## Abstract

Carbapenem resistance in *Klebsiella pneumoniae* is important because of its increasing prevalence and limited therapeutic options. To investigate the clinical and microbiological characteristics of patients infected or colonized with carbapenem non-susceptible *K. pneumoniae* (CnsKP) in Taiwan, we conducted a retrospective study at Taipei Veterans General Hospital from January 2012 to November 2013. Carbapenem non-susceptibility was defined as a minimum inhibitory concentration (MIC) of ≥2 mg/L for imipenem or meropenem. A total of 105 cases with CnsKP were identified: 49 patients with infection and 56 patients with colonization. Thirty-one isolates had genes that encoded carbapenemases (29.5%), including *K. pneumoniae* carbapenemase (KPC)-2 (n = 27), KPC-3 (n = 1), VIM-1 (n = 1) and IMP-8 (n = 2). The in-hospital mortality among patients with CnsKP was 43.8%. A MIC for imipenem ≥16 μg/mL, nasogastric intubation and Acute Physiology and Chronic Health Evaluation II score were independent risk factors for in-hospital mortality for all patients with CnsKP. A MIC for imipenem ≥16 μg/mL was also an independent risk factor for 14-day mortality in patients with CnsKP. In conclusion, a positive culture for CnsKP was associated with high in-hospital mortality. A high imipenem MIC of CnsKP can predispose a patient to a poor prognosis.

*Klebsiella pneumoniae* is an important causative agent of nosocomial and community-acquired Gram-negative bacteremia. It can cause various infections, including pneumonia, urinary tract infections and intra-abdominal infections^1^,^2^. *K. pneumoniae* is also the major pathogenic organism of community-onset pyogenic infection in Taiwan and other Asian countries^3^,^4^,^5^,^6^. *K. pneumoniae* strains possessing extended spectrum β-lactamases (ESBL) conferring multidrug resistance have been reported worldwide. Carbapenems have been used extensively for severe infections arising from ESBL-producing *Enterobacteriaceae*, thereby imposing selection pressure for carbapenem resistance^7^. One of the major carbapenem resistance mechanisms of *K. pneumoniae* is the acquisition of carbapenemase genes that encode enzymes that are able to hydrolyze carbapenems. Another mechanism is the deficiency of outer-membrane porin expression with an overexpression of β-lactamases that possess very weak affinity for carbapenems^8^.

Over the past decade, the prevalence of carbapenem resistance among *K. pneumoniae* has increased dramatically worldwide^9^. The mortality attributable to carbapenem-resistant *K. pneumoniae* infection varies between 26% and 44%, with the highest mortality reported in patients with bacteremia^10^. Studies focusing on carbapenem-resistant *K. pneumoniae* have derived primarily from carbapenemase-producing *K. pneumoniae*, especially *K. pneumoniae* carbapenemase (KPC)-producing *K. pneumoniae*^7^,^10^. The features of patients with non-carbapenemase producing carbapenem-resistant *K. pneumoniae* have rarely been reported^11^. In clinical practice, distinguishing colonization from infection is difficult, especially when isolates are collected from nonsterile sites such as the respiratory tract, urine or wounds. The clear features and outcomes of patients (whether infection or colonization) from whom carbapenem-resistant *K. pneumoniae* was isolated, regardless of the resistance mechanisms, have also rarely been reported.

To address this gap in the knowledge, we conducted an observational study to investigate the clinical and microbiological features of patients with carbapenem non-susceptible *K. pneumoniae* (CnsKP) in Taiwan. Our attention was particularly focused on the mortality related to the minimum inhibitory concentration (MIC) levels of imipenem.

## Methods

### Setting, study design and patients

This retrospective study was conducted at Taipei Veterans General Hospital (a 2900-bed and tertiary-care teaching hospital in Taiwan) from January 2012 to November 2013. All isolates were from clinical cultures during the study period. Surveillance cultures were not conducted during this period. During this 23-month study period, clinical information was collected from all consecutive patients in whom different specimens revealed isolated *K. pneumoniae* that showed non-susceptibility to carbapenem. Resistance to carbapenem was defined as a MIC of ≥2 mg/L for imipenem or meropenem according to the interpretative criteria from the Clinical and Laboratory Standards Institute (CLSI) guidelines^12^. Patients under the age of 20 and those with incomplete medical records were excluded. The protocol was approved by the Institution Review Board of Taipei Veterans General Hospital, and informed consent was waived by Institutional Review Board.

### Definitions

Infection or colonization and the probable infectious source were determined on the basis of microbiological results and on the judgments of two infectious disease specialists. The infection and colonization of CnsKP were defined as previously described^13^. In summary, an infection was confirmed by a positive culture for CnsKP isolated from blood or any other sterile source. Positive respiratory cultures were defined according to the criteria by the American Thoracic Society and the Infectious Diseases Society of America^14^. The criteria outlined by the Centers for Disease Control and Prevention National Healthcare Safety Network were used for patients with positive cultures from urine or surgical wounds^15^. Positive cultures for CnsKP from non-surgical wounds were deemed to be infected only if infection was documented in their medical records. All other culture episodes were defined as colonizations. Isolates from index cultures that had been collected more than 48 h after admission to hospital were defined as “hospital-acquired”. The isolates were defined as “healthcare-associated” if the patients lived in a nursing home or long-term care facility, had ever been admitted to a hospital for ≥2 days during the previous 90 days, had ever undergone renal dialysis in a hospital or clinic during the previous 30 days or had ever received intravenous therapy at home or in an outpatient clinic during the previous 30 days^3^. The Acute Physiology and Chronic Health Evaluation II (APACHE II) score at the time of onset of infection was used to assess the severity of illness, as described previously^16^. For patients with colonization by CnsKP, the APACHE II score was also calculated within 24 h before the positive cultures. An “immunocompromised” condition included the presence of neutropenia, human immunodeficiency virus infection or steroid therapy (≥20 mg of prednisone or equivalent per day for ≥1 month) or other immunosuppressive therapy during the 30 days before the isolation of CnsKP. Treatment with at least one antibiotic agent for ≥48 h to which the isolate was susceptible *in vitro* according to the interpretative criteria from the CLSI guidelines^12^ was defined as “appropriate therapy”. Tigecycline was considered “appropriate therapy” for bloodstream or urinary tract infection if the tigecycline MIC was ≤0.5 mg/L with standard dosing^17^.

### Predictors of mortality

We compared the clinical variables between survivors and non-survivors to investigate the risk factors for mortality in patients with CnsKP. The variables reviewed included infection or colonization, demographic characteristics, MIC value for imipenem in CnsKP, history of various underlying diseases, immunocompromised state, presence of indwelling devices at the time of CnsKP isolation, any type of surgery in the previous 30 days, APACHE II score, length of stay in the intensive care unit (ICU) at the time of isolation of CnsKP and previous hospitalization. The appropriate use of antibiotics was analyzed in patients with infection.

### Microbiologic methods

The identification and antimicrobial susceptibility testing of bacterial strains was performed using a Vitek 2 automated system (bioMérieux, Marcy l’Etoile, France). MICs were classified according to the CLSI breakpoints^12^, except those for colistin and tigecycline. For colistin, susceptibility was defined based on the European Committee on Antimicrobial Susceptibility Testing (susceptible, MIC ≤ 2 mg/L; resistant, MIC > 2 mg/L) as described previously^18^. Susceptibility to tigecycline was defined according to the recommendation by the US Food and Drug Administration (FDA) (susceptible, MIC ≤ 2 mg/L; resistant, MIC ≥ 8 mg/L), as described previously^18^.

Polymerase chain reaction (PCR) was used for all isolates to detect carbapenemase genes (encoding Ambler class-A families NMC, IMI, SME, KPC and GES; Ambler class-B families IMP, VIM, GIM, SIM, SPM and NDM; and Ambler class-D family OXA-48-type), ESBL genes (encoding CTX-M, SHV and TEM) and plasmid-borne AmpC-like genes (encoding CMY and DHA) as described previously^18^,^19^. Identification of outer-membrane porins (OMPK35 and OMPK36) was carried out as described previously^18^.

### Statistical analyses

Discrete variables were analyzed using the chi-square test or Fisher’s exact test. Continuous variables were compared using Student’s *t*-test or Mann–Whitney rank sum test. The independent risk factors for 14-day and in-hospital mortality were explored by logistic regression models. Univariate analyses were performed separately for each risk-factor variable to determine the odds ratio (OR) and 95% confidence interval (CI). All biologically plausible variables with *p* ≤ 0.20 in the univariate analysis were included in the logistic regression model for the multivariate analysis. A backward selection process was utilized.

## Results

### Microbiological characteristics of CnsKP isolates

During the study period, 105 patients had isolates of a CnsKP strain in the following clinical samples: sputum (n = 41), pleural effusion (n = 1), urine (n = 39), central venous catheter tip (n = 3), blood (n = 10), wound (n = 6) and bile (n = 5). Thirty-one isolates (29.5%) had genes that encoded carbapenemases, including KPC-2 (n = 27), KPC-3 (n = 1), VIM-1 (n = 1) and IMP-8 (n = 2). The most common mechanism of carbapenem resistance, as identified in 74 cases, was the production of AmpC-mediated β-lactamases or ESBL plus porin defects. Among the 74 isolates, 55 exhibited Amp-C β-lactamase (all DHA-1). Among the isolates with genes encoding ESBLs, 38 isolates exhibited the CTX-M-9 group, and 12 isolates exhibited the CTX-M-1 group. Forty-nine isolates harbored the SHV-type ESBL genes (SHV-2, SHV-5, SHV-12, SHV-31 and SHV-120). The clinical data suggested no epidemiological links between these isolates.

The MIC ranges, MIC_50_ values and MIC_90_ values of various antimicrobial agents against the CnsKP isolates are listed in Table 1. The MIC of imipenem was ≥16 μg/mL for 44 (41.9%) isolates, 8 μg/mL for 18 (17.1%) isolates, 4 μg/mL for 23 (21.9%) isolates and 2 μg/mL for 19 (18.6%) isolates. Most isolates were susceptible to colistin (n = 93, 88.6%), tigecycline (n = 86, 81.9%), and amikacin (n = 84, 80%), but they showed only moderate susceptibility to gentamicin (n = 59, 56.2%).

### Characteristics of patients with CnsKP

A total of 105 cases with CnsKP were identified during the study period. The 14-day mortality associated with CnsKP was 20%. The overall in-hospital mortality associated with CnsKP was 43.8%. Infection was diagnosed in 49 patients, and colonization was established in the remaining 56 patients. The 14-day mortality was significantly higher in patients with infection than in those with colonization (34.7% *vs*. 7.1%, *p* < 0.001). The overall in-hospital mortality was also significantly higher in patients with infection than in those with colonization (63.3% *vs*. 26.8%, P < 0.001).

We further compared the clinical features between patients infected with CnsKP and those colonized with CnsKP (Table 2). Patients with infection had a greater prevalence of fatal outcomes than did those without infection. The Charlson Comorbidity Index and APACHE II scores were significantly higher for patients with infection than for those with colonization. Patients with colonization had a higher rate of previous antimicrobial therapy than did those with infection. For the antimicrobial susceptibility profiles, the prevalence of imipenem MIC ≥ 16 μg/mL tended to be higher in the infection group (51.0%) than in the colonization group (33.9%) (*p* = 0.077).

### Risk factors for mortality in patients with CnsKP

To clarify the influence of clinical and microbiological factors on both 14-day and in-hospital mortality, multivariate logistic regression analyses were conducted for the entire cohort to identify independent risk factors for mortality (Tables 3 and 4). All biologically plausible variables with *p* ≤ 0.20 in the univariate analysis were considered for inclusion in the logistic regression model for multivariate analysis. Septic shock, appropriate antibiotics and clinical syndromes were not included because we enrolled patients with infection and colonization. Among the 105 patients, isolation of a CnsKP with imipenem MIC ≥ 16 μg/mL (OR, 3.17; 95% CI, 1.11–9.05; *p* = 0.032) and APACHE II score (OR, 1.24; 95% CI, 1.13–1.36; *p* < 0.001) were independent risk factors for 14-day mortality (Table 3). Isolation of a CnsKP with imipenem MIC ≥ 16 μg/mL (OR, 5.60; 95% CI, 1.39–22.50; *p* = 0.015), nasogastric intubation (OR, 6.19; 95% CI, 1.07–9.05; *p* = 0.042) and APACHE II score (OR, 1.13; 95% CI, 1.06–1.21; *p* < 0.001) were independent risk factors for in-hospital mortality (Table 4).

We further evaluated the 49 patients infected with CnsKP. The 14-day mortality for cases with infections was 34.7% (17/49). All biologically plausible variables with *P* ≤ 0.20 in the univariate analysis were considered for inclusion in the logistic regression model for multivariate analysis. Multivariate logistic regression analyses showed that MIC for imipenem ≥16 μg/mL (OR, 21.75; 95% CI, 1.47–321.55; *p* = 0.025), bacteremia (OR, 15.09; 95% CI, 1.24–183.56; *p* = 0.033) and APACHE II score (OR, 1.35; 95% CI, 1.10–1.65; *p* = 0.005) were independent risk factors for 14-day mortality (Table 5).

## Discussion

The current study is one of the largest series of infection or colonization of CnsKP in adult patients. Carbapenemase producers accounted for ≈29.5% of the CnsKP isolates. The 14-day mortality among patients with CnsKP was 20%. The overall in-hospital mortality associated with CnsKP was 43.8%. A MIC for imipenem ≥16 μg/mL was an independent risk factor for both 14-day and in-hospital mortality for all patients with CnsKP.

The literature suggests that patients with carbapenem-resistant *Enterobacteriaceae* (CRE) isolated from any site, regardless of whether infection exists, are associated with poor outcomes^20^. Won *et al*. reported that the odds of fatal outcomes for patients with infection were higher than the odds for those with colonization with regard to isolation with KPC-producing *Enterobacteriaceae* (OR, 3.8), but the difference was not significant^21^. Consistent with the literature, we found high in-hospital mortality among patients with CnsKP, regardless of infection or colonization. We also found that both 14-day and in-hospital mortality were significantly higher in infected patients than in those colonized with CnsKP. This finding suggests that positive cultures for CnsKP in clinical samples represent an emerging challenge for physicians.

It is notable that non-carbapenemase-mediated mechanisms account for the majority of *in vitro* resistance mechanisms responsible for CnsKP in Taiwan, which is consistent with previous results^18^. It is difficult to distinguish between infection and colonization if CRE isolates are obtained from non-sterile sites in clinical practice. Therefore, the current investigation evaluated the detailed clinical features of all patients from whom CnsKP was isolated. The current study found that more than half of the patients were colonized with CnsKP. The higher Charlson Comorbidity Index among patients with infection suggested the opportunistic nature of CnsKP and that underlying comorbidity played a major role in CnsKP infection. We found that the infection and colonization groups had both had heavy exposure to antibiotics within the previous month. This finding might suggest that antimicrobial stewardship is important in the control of CnsKP.

The significance of positive cultures for CnsKP in clinical samples is not addressed clearly. In this study, we found that positive culture for CnsKP was associated with high in-hospital mortality, regardless of colonization or infection. Because both colonization and infection were common problems in clinical practice, we tried to analyze the risk factors for mortality among these patients. In the literature, the risk factors for mortality in all patients with positive cultures for CnsKP have never been reported^7^,^20^. Several studies have indicated that patients who were infected by Gram-negative bacteria with higher carbapenem MICs and who received carbapenems were associated with poorer clinical outcomes^22^,^23^,^24^. Compared to previous studies, we tried to analyze all patients with positive cultures of CnsKP in this study regardless of infection or colonization. We were the first to demonstrate that an imipenem MIC of ≥16 μg/mL was an independent risk factor for both 14-day and in-hospital mortality among these patients. This result suggested that a positive culture for CnsKP with an imipenem MIC ≥16 μg/mL in clinical samples is of clinical significance and may suggest that the resistance level of carbapenem is as important as the presence of carbapenemase among CnsKP. It also displays that isolation of CnsKP with a high MIC value is a marker of severe underlying conditions that are associated with poor outcomes. High MIC values would also predict the limited efficacy of those drugs against CnsKP infections. Given the notable predictor for mortality observed among CnsKP isolates with high MIC values, these data suggest that MIC may play an important part in predicting outcome. One recent study suggested that antimicrobial MICs and/or molecular markers of resistance may be useful in identifying the strains that are most likely to respond to some types of antibiotics^25^. These findings imply that the microbiological features of CnsKP play an important role in clinical outcomes.

The limitations of our study are its inherently retrospective design and the relatively limited number of occurrences of this disease. The limited numbers of cases also reduced the power of the analysis of risk factors for mortality. Evaluation of the relative efficacy of appropriate antibiotics in cases with infection was limited because of the small number of patients. Validation of our findings requires future studies at other centers and in larger populations. In addition, we tried to address the significance of positive cultures for CnsKP in clinical samples in this study. However, periodical surveillance cultures were not performed among these patients. Therefore, whether a case with CnsKP infection had previous CnsKP colonization was not clear.

In conclusion, the major mechanism of the CnsKP phenotype in Taiwan was the deficiency of outer-membrane porins in association with β-lactamases such as the AmpC enzyme or ESBLs. A positive culture for CnsKP was associated with high in-hospital mortality. We were the first to demonstrate that an imipenem MIC of ≥16 μg/mL was an independent risk factor for both 14-day and in-hospital mortality among patients with CnsKP. Clinicians should thus consider the imipenem MIC when they manage cases with CnsKP.

## Additional Information

**How to cite this article**: Wu, P.-F. *et al*. High minimum inhibitory concentration of imipenem as a predictor of fatal outcome in patients with carbapenem non-susceptible *Klebsiella pneumoniae*. *Sci. Rep.* **6**, 32665; doi: 10.1038/srep32665 (2016).

## Figures and Tables

**Table 1:** *In vitro* activities of the antimicrobial agents tested against 105 CnsKP isolates.

Antimicrobial agent	MIC range (μg/mL)	MIC_50_^a^ (μg/mL)	MIC_90_^b^ (μg/mL)	No. (%) of isolates susceptible
Ciprofloxacin	≤0.25 to ≥4	≥4	≥4	4 (3.8)
Levofloxacin	≤0.12 to ≥8	≥8	≥8	4 (3.8)
Ampicillin-sulbactam	≥32	≥32	≥32	0 (0)
Piperacillin-tazobactam	≥128	≥128	≥128	0 (0)
Cefazolin	≥64	≥64	≥64	0 (0)
Ceftriaxone	2 to ≥64	≥64	≥64	0 (0)
Ceftazidime	16 to ≥64	≥64	≥64	0 (0)
Cefepime	≤1 to ≥64	≥64	≥64	12 (11.4)
Amikacin	≤2 to ≥64	≤2	≥64	84 (80)
Gentamicin	≤1 to ≥16	≤1	≥16	59 (56.2)
Ertapenem	2 to ≥8	≥8	≥8	0 (0)
Imipenem	1 to ≥16	8	≥16	1 (0.9)
Colistin	≤0.5 to ≥16	≤0.5	2	93 (88.6)
Tigecycline	≤0.5 to ≥8	2	4	86 (81.9)

CnsKP: carbapenem non-susceptible *Klebsiella pneumoniae*; MIC: minimum inhibitory concentration.

^a^MIC_50_: MIC for 50% of isolates.

^b^MIC_90_: MIC for 90% of isolates.

**Table 2:** Characteristics of patients with CnsKP infection or colonization.

Variable	CnsKP infection (n = 49)	CnsKP colonization (n = 56)	*p*
Demographics			
Age, years, mean ± SD	79.7 ± 12.0	75.8 ± 14.7	0.140
Male sex	33 (67.3)	39 (69.6)	0.800
Nosocomial-acquired isolate	37 (75.5)	48 (85.7)	0.184
Microbiological characteristics of CnsKP			
Carbapenemase-producing isolates	17 (34.7)	14 (25.0)	0.277
Imipenem MIC ≥4	39 (79.6)	46 (82.1)	0.740
Imipenem MIC ≥8	30 (61.2)	32 (57.1)	0.671
Imipenem MIC ≥16	25 (51.0)	19 (33.9)	0.077
Polymicrobial isolation	22 (44.9)	19 (33.9)	0.250
Comorbidities			
Diabetes mellitus	27 (55.1)	23 (41.1)	0.151
COPD	33 (67.3)	31 (55.4)	0.724
Heart failure	14 (28.6)	16 (28.6)	1.000
Cerebrovascular disease	16 (32.7)	11 (19.6)	0.128
Chronic renal failure	18 (58.1)	46 (62.2)	0.695
Liver cirrhosis	2 (4.1)	5 (8.9)	0.321
Malignancy	16 (32.7)	11 (19.6)	0.128
Immunocompromised state	8 (16.3)	13 (23.2)	0.379
Charlson Comorbidity Index, mean ± SD	4.9 ± 2.5	3.6 ± 2.5	0.009
Indwelling central venous catheter	20 (40.8)	18 (32.1)	0.356
Indwelling urinary catheter	31 (63.3)	34 (60.7)	0.788
Nasogastric tube	38 (77.6)	42 (75.0)	0.759
Surgical drainage	8 (16.3)	11 (19.6)	0.660
Mechanically ventilated at isolation	17 (34.7)	14 (25.0)	0.277
Renal dialysis at isolation	13 (26.5)	8 (14.3)	0.118
Previous surgery^a^	15 (30.6)	16 (28.6)	0.819
Prior use of antimicrobials^b^			
Any	43 (87.8)	55 (98.2)	0.048
β-lactams plus β-lactamase inhibitors	13 (26.5)	19 (33.9)	0.411
Fluoroquinolones	20 (40.8)	26 (46.4)	0.563
Carbapenems	20 (40.8)	28 (50.0)	0.346
Third- and fourth-generation cephalosporins	19 (38.8)	20 (35.7)	0.746
Glycopeptides	20 (40.8)	16 (28.6)	0.187
Aminoglycosides	1 (2.0)	2 (3.6)	1.000
Metronidazole	7 (14.3)	8 (14.3)	1.000
Colistin	10 (20.4)	11 (19.6)	0.922
Tigecycline	15 (30.6)	11 (19.6)	0.194
APACHE II score, mean ± SD	25.4 ± 9.4	17.6 ± 8.2	<0.001
14-day mortality^c^	17 (34.7)	4 (7.1)	<0.001
In-hospital death	31 (63.3)	15 (26.8)	<0.001

Data are the number (%) unless specified otherwise.

APACHE, Acute Physiology and Chronic Health Evaluation; BSI, bloodstream infection; CI, confidence interval; CnsKP: carbapenem non-susceptible *Klebsiella pneumoniae*; COPD, chronic obstructive pulmonary disease; IQR, interquartile range; LOS, length of hospital stay; OR, odds ratio; SD, standard deviation.

^a^During the 3 months preceding BSI onset.

^b^During the 30 days preceding BSI onset.

^c^Death within 14 days of the isolation of CnsKP.

**Table 3:** Logistic regression analysis of predictors for 14-day mortality among 105 patients with CnsKP isolates.

Variable	Univariate analysis		Multivariable analysis	
	OR (95% CI)	*p*	OR (95% CI)	*p*
Infection	6.90 (2.13–22.36)	0.001		
Carbapenemase-producing CnsKP	2.73 (1.02–7.33)	0.047		
Imipenem MIC ≥4	5.85 (0.74–46.44)	0.095		
Imipenem MIC ≥8	2.64 (0.89–7.88)	0.081		
Imipenem MIC ≥16	3.60 (1.31–9.90)	0.013	5.60 (1.39–22.50)	0.015
Diabetes mellitus	2.06 (0.77–5.50)	0.147		
Chronic renal failure	2.40 (0.86–7.66)	0.117		
Charlson Comorbidity Index	1.15 (0.96–1.39)	0.136		
Indwelling central venous catheter	3.84 (1.42–10.40)	0.008		
Nasogastric tube	3.58 (0.77–16.60)	0.103		
Mechanically ventilated at isolation	2.73 (1.02–7.33)	0.047		
Renal dialysis at isolation	2.50 (0.86–7.31)	0.094		
APACHE II score	1.21 (1.12–1.31)	<0.001	1.24 (1.13–1.36)	<0.001

Data are the number (%) unless specified otherwise.

APACHE, Acute Physiology and Chronic Health Evaluation; CI, confidence interval; CnsKP: carbapenem non-susceptible *Klebsiella pneumoniae*; OR, odds ratio.

**Table 4:** Logistic regression analysis of predictors for in-hospital mortality among 105 patients with CnsKP isolates.

Variable	Univariate analysis		Multivariable analysis	
	OR (95% CI)	*p*	OR (95% CI)	*p*
Infection	6.90 (2.13–22.36)	0.001		
Nosocomial-acquired isolates	2.80 (0.93–8.38)	0.066		
Imipenem MIC ≥4	5.80 (1.58–21.27)	0.008		
Imipenem MIC ≥8	2.21 (0.98–4.96)	0.055		
Imipenem MIC ≥16	3.51 (1.56–7.92)	0.002	3.17 (1.11–9.05)	0.032
Diabetes mellitus	2.06 (0.87–4.14)	0.108		
Chronic renal failure	2.74 (1.19–6.30)	0.018		
Charlson Comorbidity Index	1.31 (1.11–1.56)	0.002		
Indwelling central venous catheter	2.93 (1.29–6.68)	0.010		
Nasogastric tube	8.52 (2.36–30.77)	0.001	6.19 (1.07–9.05)	0.042
Mechanically ventilated at isolation	4.12 (1.68–10.06)	0.002		
Renal dialysis at isolation	2.14 (0.80–5.73)	0.131		
APACHE II score	1.17 (1.10–1.25)	<0.001	1.13 (1.06–1.21)	<0.001

Data are the number (%) unless specified otherwise.

APACHE, Acute Physiology and Chronic Health Evaluation; CI, confidence interval; CnsKP: carbapenem non-susceptible *Klebsiella pneumoniae*; OR, odds ratio.

**Table 5:** Logistic regression analysis of predictors for 14-day mortality among 49 patients infected with isolation of CnsKP.

Variable	Univariate analysis		Multivariable analysis	
	OR (95% CI)	*p*	OR (95% CI)	*p*
Carbapenemase-producing CnsKP	2.27(0.67–7.73)	0.189		
Imipenem MIC ≥4	6.26(0.72–54.41)	0.096		
Imipenem MIC ≥8	2.87 (0.77–10.77)	0.117		
Imipenem MIC ≥16	3.51(0.99–12.36)	0.051	21.75 (1.47–321.55)	0.025
Pneumonia	3.65(1.06–12.56)	0.040		
Urinary tract infection	0.31(0.08–1.31)	0.112		
Bacteremia	3.81(0.90–16.19)	0.069	15.09(1.24–183.56)	0.033
Septic shock	8.57(2.25–32.68)	0.002		
Polymicrobial infection	0.37(0.11–1.29)	0.118		
Mechanically ventilated at isolation	2.27(0.67–7.73)	0.189		
APACHE II score	1.21(1.08–1.36)	0.001	1.35(1.10–1.65)	0.005
Appropriate antibiotics use	0.37(0.11–1.26)	0.113		

Data are the number (%) unless specified otherwise.

APACHE, Acute Physiology and Chronic Health Evaluation; CI, confidence interval; CnsKP: carbapenem non-susceptible *Klebsiella pneumoniae*; OR, odds ratio; SD, standard deviation.
